# Micro Sensor Node for Air Pollutant Monitoring: Hardware and Software Issues

**DOI:** 10.3390/s91007970

**Published:** 2009-10-12

**Authors:** Sukwon Choi, Nakyoung Kim, Hojung Cha, Rhan Ha

**Affiliations:** 1 Department of Computer Science, Yonsei University, Seodaemungu, Shinchondong, Seoul, Korea; E-Mails: sukwon@cs.yonsei.ac.kr (S.C.); nykim@cs.yonsei.ac.kr (N.K.); 2 Department of Computer Engineering, Hongik University, Mapogu, Sangsoodong, Seoul, Korea; E-Mail: rhanha@cs.hongik.ac.kr

**Keywords:** gas sensors, wireless sensor networks, air pollutant monitoring

## Abstract

Wireless sensor networks equipped with various gas sensors have been actively used for air quality monitoring. Previous studies have typically explored system issues that include middleware or networking performance, but most research has barely considered the details of the hardware and software of the sensor node itself. In this paper, we focus on the design and implementation of a sensor board for air pollutant monitoring applications. Several hardware and software issues are discussed to explore the possibilities of a practical WSN-based air pollution monitoring system. Through extensive experiments and evaluation, we have determined the various characteristics of the gas sensors and their practical implications for air pollutant monitoring systems.

## 1. Introduction

The construction of good air quality systems has recently been a focus of attention with increasing reports of health problems related to poor atmospheric conditions. Detecting pollutants in the air and determining polluted areas using an air monitoring system is important as the initial process of common air-quality improvement techniques such as source control, improved ventilation, and air cleaning [1].

Conventional air quality monitoring approaches such as gas chromatography (GC) are limited with respect to time, expense, and installation sites. Therefore, limited data is available for the estimation of ambient air toxins. Further, air quality monitoring systems built into compact, handheld devices have spatial and temporal limitations, since the measurements are conducted manually [2]. Recent advances in micro-electro-mechanical systems (MEMS) and wireless sensor network (WSN) technology have allowed the creation of a low-cost air pollutant monitoring system and its deployment in real environments. The integration of an air pollutant monitoring system with WSN technology will reduce installation costs and enable the quick and easy reconfiguration of the data acquisition and control systems. In addition, networked air pollutant monitoring allows continuous and low-cost observation.

Several recent studies have applied WSN technology to air quality monitoring systems. Typically, the projects have focused on developing middleware, data aggregation and networking techniques for large-scale networks [3-5]. However, few studies have been found in the literature to deal with practical development issues related to the sensor node itself, which consists of various types of MEMS-based or infrared-based micro gas sensors. Although many of the recently developed gas sensors exhibit small form factors, their operation still consumes a nontrivial amount of energy, and their sensing accuracy needs improvement [6]. Therefore, a WSN-specific issue, particularly when dealing with diverse gas sensors, should be studied in terms of both hardware- and software-related aspects.

In this paper, we present the design and implementation of the APOLLO (Air POLLutants mOnitoring system) sensor node, which is constructed with off-the-shelf MEMS-based or infrared-based micro gas sensors. APOLLO provides air quality information by collecting independent sensing information from various air components and forwarding the collected data to the host system. When implementing the system, several issues should be addressed, in terms of both hardware and software. We present some of the design factors necessary to choose the right set of gas sensors, their performance and power-consumption characteristics, and various software development issues. The sensors are extensively evaluated both in performance and power management aspects.

This paper is organized as follows: Section 2 presents related work. The design goals of the proposed system are discussed in Section 3. The hardware and software parts of the system are discussed in Section 4 and Section 5, respectively. Section 6 describes the implementation detail of APOLLO. Section 7 evaluates the proposed system, and is followed by general discussion in Section 8. Section 9 concludes the paper.

## 2. Related Work

Previous research has attempted to construct networked air quality-monitoring and diagnosis systems. SensorScope [7] and CitySense [8] are examples of large-scale wireless environmental monitoring systems. SensorScope was developed to provide *in-situ* spatial and temporal observations across the landscape. CitySense supports the development and evaluation of wireless systems that span an entire city by employing over 100 Wi-Fi enabled Linux-based PCs embedded throughout buildings and streetlights. While SensorScope makes use of solar energy with extensive radio duty cycling to prevent power outages, CitySense uses a wired power supply. Other systems have also been developed on energy-intensive platforms. For example, N-SMARTS [9] is a GPS-enabled cell phone-based or car-mounted citywide environmental data acquisition system. Its sensor module consists of carbon dioxide, carbon monoxide, three-axis accelerometer, and temperature sensors. SensorMap [10] is a mobile air quality-monitoring network comprised of sensors that can detect O_3_, NO_2_ and CO/VOC. The scheme focused on data collection and presentation, but did not consider issues like the characteristics of the gas sensors and energy management. LaserSPECks [11] was developed based on a laser spectroscopic trace-gas sensor platform. By integrating quantum cascade laser technology, both the size and cost of the system are reduced, while providing a wide range of detectable gases. However, the power consumption is not negligible.

As previous studies did not detail considerations related to issues such as energy consumption, sensor selection, or spatial flexibility, our work here describes in more detail the design, implementation, and operation of an air pollutant monitoring system constructed with compact gas sensors.

## 3. Design Goals

### 3.1. Air Pollution Monitoring for EPA-specified Criteria Pollutants

The U.S. Environmental Protection Agency (EPA) designates a standardized air pollution level indicator, the Air Quality Index (AQI), which mainly consists of carbon monoxide (CO), nitrogen dioxide (NO_2_), particulate matter (PM), carbon dioxide (CO_2_) and sulfur dioxide (SO2) [1]. A monitoring system should be able to detect these noxious gases in a timely and accurate manner with the commercial, off-the-shelf (COTS) gas sensors. For a summary of these pollutants and their effects on human body, see Table 1. The primary goal of our system is to build an air quality-monitoring tool that measures the EPA-specified criteria pollutants with inexpensive compact sensors.

### 3.2. Flexibility and Energy Management

Air pollution can be generated in any environment without the distinction of indoor or outdoor sources, including streets, schools, offices, houses, subway stations, mines, and so forth. Conventional air condition monitoring approaches such as gas chromatography and passive sampling methods are limited in terms of their installation cost, time, and size. Therefore, an air quality-monitoring system should be spatially flexible and straightforward to monitor the air quality of a target area. Furthermore, the system should function with various power supplies such as battery, AC, or DC, since power outlets are sometimes available at the monitoring site. In addition, the cost of sensor instruments should be reasonable, so that multiple sensors can be installed to effectively sense an area online.

Most of the existing WSN-based air quality-monitoring systems rely on stationary node deployment and an always-available power supply. However, battery-powered sensor nodes provide higher spatial flexibility in comparison to a system that uses static power outlets. Since sensor nodes are freely positioned in the site, the system can be deployed and removed quickly and easily. Hence, the system can operate in emergencies such as earthquakes, fires, brownouts, or blackouts in which electric power supplies are disrupted. In addition, optimal installation positions can be straightforwardly located by considering the airflows of the target area, which enables the precise and accurate assessment of air quality. The power management of the battery-operated sensor nodes is essential to maximizing the system's lifetime. Various sensors are required to construct an air monitoring system, and some gas sensors based on chemical reactions consume a significant amount of energy compared to other kinds of sensors. Therefore, the appropriate power management of the sensor nodes should be conducted.

## 4. Hardware Design

### 4.1. GAS Sensors

Before providing a detailed explanation of each component of APOLLO, we first introduce the principles and characteristics of the chemical gas sensors mounted on our sensor board. Several types of COTS chemical gas sensors exist, but each sensor has different operation principles. The operational characteristics of a gas sensor are generally classified into three types: heating semiconductor, non-dispersive infrared (NDIR), and light emitting diode (LED). The size, accuracy, and power consumption of a compact gas sensor all vary with sensor type.

The heating semiconductor sensor evaluates a target gas concentration by measuring the electrical conductivity of a sensing layer that is composed of a metal-oxide material such as tin dioxide (SnO_2_) or zinc oxide (ZnO). When toxic gases reach the sensor's surface and are absorbed, its electrical conductivity changes. For semiconductor sensors, a warm-up time is needed because the semiconducting oxides react sensitively to vapor and other chemicals. For example, the output of the CO sensor, MiCS-5521, is stabilized when the temperature reaches about 340 °C. Pre-heating eliminates both vapor and impurities on the sensing layer so that the chemical equilibrium is achieved. With the use of MEMS technology, this type of sensor is small in size and has a fast response time [6]. The CO, NO_2_, and VOC sensors belong to this category.

The NDIR sensor consists of an infrared lamp, a sample chamber or light tube, a wavelength filter, and the infrared detector. Gas is pumped into the sample chamber, and the gas concentration is measured electro-optically by absorbing a specific wavelength in infrared. The infrared light is directed through the sample chamber towards the detector, which has an optical filter in front of it to eliminate all light except the wavelength absorbable by the selected gas molecules. Ideally, other gas molecules do not absorb light at this wavelength and do not affect the amount of light reaching the detector. NDIR sensors usually consume more energy than the semiconductor sensors; however, they provide accurate measurements. The CO_2_ sensor belongs to this category.

An LED sensor such as the PPDNS4 counts the number of particles based on the amount of LED light a particle blocks when passing through the detection area of the sensor. Since this type of sensor has a heater for air circulation inside the sensor, its power consumption is significantly higher than that of the other sensors.

### 4.2. Sensor Board

Since the primary goal of APOLLO is to provide information about the EPA-specified criteria pollutants using inexpensive compact sensors, we used COTS gas sensors that would satisfy the requirements of low cost and applicability. The chosen sensors, which detect CO, CO_2_, NO_2_, particulate matter (PM), and volatile organic compounds (VOCs), were integrated into a single board for analysis of the characteristics of each sensor in a consistent environment. The VOC sensor was selected because it detects SO_2_. A temperature/humidity sensor was also mounted on the sensor board, since the sensing results of the gas sensors are sensitive to ambient temperature and humidity.

Mounting several gas sensors on a single integrated sensor board has advantages in terms of energy use, cost, installation time, and pollutant detection compared to multiple and separate sensor board designs. As described in Table 1, many noxious gases are generated from the same contamination source. Therefore, placing the sensors close together enables the valid detection of air pollutants. The accuracy of the semiconductor gas sensors is normally influenced by temperature and humidity; therefore, a considerable baseline drift may be caused. To overcome this problem, the humidity and temperature sensors were attached to the sensor board and correction algorithms applied.

However, integrating several sensors into a sensor board presented a series of unexpected problems in terms of power supply. Table 2 shows that the rated voltages of the six sensors differ significantly.

In an early design, we used a voltage boosting circuit that enables 12-V supply from the 3-V power source. This method caused a critical voltage drop, and, consequently, the radio transceiver did not function properly. In addition, the sensor node consumed two AA batteries after about one hour of operation. With the hardware revision, the power supply was separated from the sensor node and changed to a 12-V rechargeable battery. To support the three kinds of rated voltage, two DC-DC converters were employed: one for step-down 12 V to 3 V and the other for step-up 3 V to 5 V.

**Figure 1.**
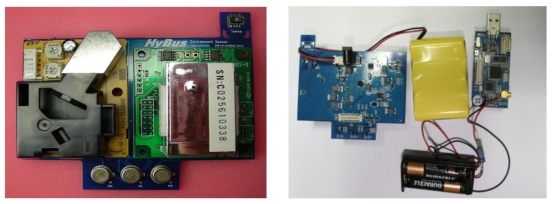
Add-on sensor board and dual power system.

After changing the power supply designs, the sensors such as the PPD4NS and D-120 still did not function properly after a certain time of operation, due to their excessive power requirements. We concluded that a continuous power supply was not feasible, even with larger batteries; hence, sensor operations should be conducted as power-manageable components. In our final design, all the gas sensors had electrical switches to enable software-based power management. The final hardware is shown in Figure 1. The sensor board was basically powered by a lithium-ion rechargeable battery, but, for high spatial flexibility and convenience, the sensor board was designed such that power supply from power outlets is also possible.

One interesting observation was that the heat from chemical sensors such as the CO, VOC, and NO_2_ sensors could greatly influence the temperature measured by the SHT11 unit. During the hardware revision, we recognized that the measured temperature from the sensor was significantly higher than the actual temperature, and the diffusion of thermal energy affected the sensing accuracy of the nearby SHT11. Therefore, in the final version the SHT11 sensor was located further away from the chemical sensors.

The energy consumption of sensor board could be further reduced by eliminating the DC-DC converters. In our prototype sensor board, two DC-DC converters were employed to provide three kinds of voltages: 3 V, 5 V, and 12 V. However, since the DC-DC converters typically have low efficiency (around 70%) [12], a large amount of energy was wasted during the voltage conversion. Therefore, the unification of rated voltages among several sensors is recommended because the DC-DC converters can be removed from the sensor board.

For the base hardware to host the integrated sensor board, an IEEE802.15.4-based sensor node [13] was used. The board basically consisted of an MSP430 MCU and TI CC2420 [14] transceiver offering a data rate of 250 kbps at 2.4 GHz. The base board provides a 51-pin connector for the add-on sensor board and to forward sensing data to the base station.

## 5. Software Issues

### 5.1. Reading the Sensors

The three types of sensors used in our work have different operating mechanisms as well as different output interfaces. Heating semiconductor sensors such as the CO, NO_2_, and VOC sensors present the sensing value as a voltage level, so the value can be read using the ADC interface. NDIR sensors such as the CO_2_ sensor present the physical gas concentration values to a serial interface; thus, accurate sensing data can be obtained without an additional converting procedure. The LED-type PM sensor requires 500 Hz hardware interruptions for 30 seconds to count the number of particles. Semiconductor sensors present an immediate response to the pollutant, although the sensing data is relatively inaccurate. NDIR and LED sensors produce physical sensing data, but they require a certain operating time to obtain a stable sensing value. Therefore, the operating frequency of the system should consider the individual characteristics of the sensors.

**Figure 2.**
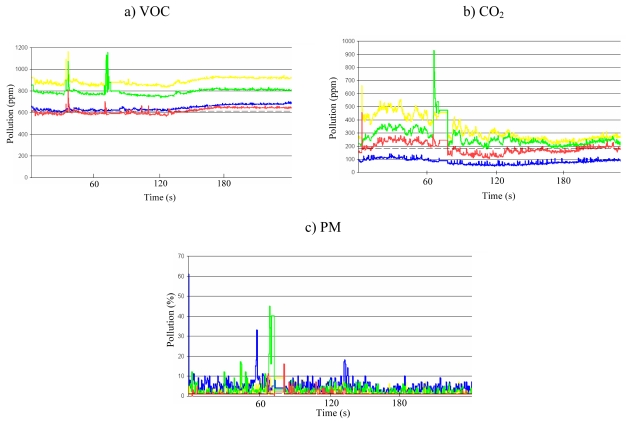
Sensing values with multiple nodes.

Preliminary experiments were conducted to understand the characteristics of each gas sensor. Figure 2 shows the preliminary experiment results obtained from the CO_2_, VOC, and PM sensors. Several sensors of the same type were used for the experiments. The experiment was performed with four different sensor nodes which were deployed at the same place with a 10 cm distance interval. Each colored line in Figure 2 represents the air pollution level for each sensor node. The graphs show that each sensor generated different sensing outputs, although the patterns of the pollution level are similar to each other, possibly due to the imprecise manufacturing process of the hardware. However, the magnitude of response to the pollutants is identical, although each sensor does not provide the same sensing values. This necessitates the calibration of sensor hardware before deployment. In fact, there have been many previous studies conducted on sensor calibration [15-18]; therefore, we did not explore this issue in detail. Our work addressed this problem simply by adjusting the baselines of the sensor nodes to a specific sensor node at the application program.

### 5.2. Power Management for Sensor Node

**Figure 3.**
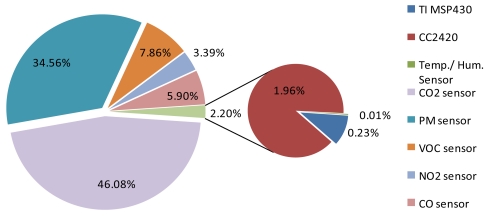
Power consumption breakdown for the sensor board and the base board.

The integration of numerous gas sensors into a single sensor board led to practical problems, especially in terms of energy. Figure 3 shows the breakdown of power consumption for our sensor board and the base node. Heating semiconductor sensors and NDIR sensors provide unreliable sensing data until the components reach the required temperature. In monitoring systems that use WSN technology and operate with a limited energy source, the sensors are basically kept off and turned on only periodically for energy-saving purposes. In the event that a monitoring system uses heating semiconductor sensors, a warm-up time must be considered when scheduling the on-off usage of sensors. Besides the warm-up time, the wake-up latency and break-even cycle of the sensor must be taken into account. Apparently, the add-on sensor board consumes a significant amount of power; therefore, appropriate power management is mandatory for the practical use of the system.

In our previous work, we developed an automated sensor-specific power management system, called ASPM (Automated Sensor-specific Power Management) [12], for wireless sensor networks. The mechanism is implemented in the RETOS operating system [19]. In ASPM, we defined essential factors for sensor-specific power management. Based on the user-provided sensor-specific information, the RETOS kernel automatically conducts the power management of hardware components including sensors. This way, it is not necessary for application programmers to develop additional code for power management.

To apply ASPM to the APOLLO system, sensor-specific characteristics such as wake-up latency, average power consumption, and break-even cycle should be evaluated through preliminary experiments. Table 3 shows the measured or calculated wake-up latencies and break-even cycles of the sensors under evaluation. ASPM cannot be directly applied to APOLLO because the system environment is different from that in our previous work.

First, the battery residual was one of the important performance factors, since the inrush currents that are generated during the transition of the sensor's power state draw a certain amount of energy. However, in APOLLO, no significant inrush current is observed in any of the chemical gas sensors on the sensor board; hence, ASPM monitors only the rated voltages of the gas sensors. Second, the battery residual of the sensor board cannot immediately be measured via the battery monitor in the CC2420 transceiver because the range of the monitor is between 1.89 V and 3.33 V, and the smallest voltage value among several sensors was 5 V. Therefore, we used a voltage sensor with a wider sensing range than the rated voltages. In all the sensors in the board, the output becomes abnormal when the input voltage is below its rated voltage. For example, the PPDNS4 sensor generates sensing values below 100 with enough power supply, whereas the output value is over 1,000 with a lack of battery capacity. Third, the battery model has also changed. We used a linear model for a specific alkaline-manganese dioxide battery, but the sensor board is powered by a Li-ion rechargeable battery. In particular, the self-discharge rate of the rechargeable battery becomes high; hence, the model should be modified appropriately.

We assumed that a user can modify the periodic behavior dynamically at runtime. Our preliminary experiments revealed that setting the application's period shorter than 180 seconds caused a severe reduction in the system's lifetime. This occurred because the chemical sensors typically require long wake-up latencies, as shown in Table 3; hence, the chemical gas sensors are turned on continuously. We addressed this problem by exploiting the *pm_helper* API of ASPM and the pulsed mode of semiconductor gas sensors such as the CO, NO_2_, and VOC sensors. If these sensors operate in the pulsed mode, they are powered periodically since the radio transceiver is duty-cycled. As a result, the wake-up latency is decreased, as the temperature of the sensing resistor will be maintained in a warm-up state.

## 6. Implementation

**Figure 4.**
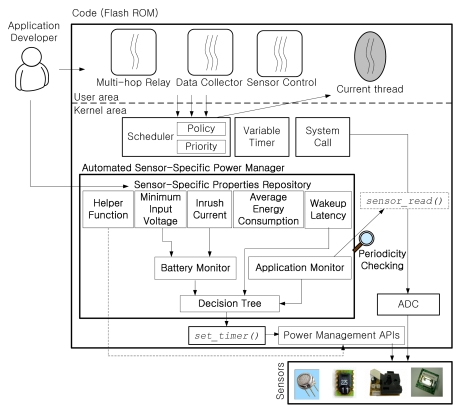
Software architecture of APOLLO on RETOS.

We developed application software running on the sensor node based on RETOS [19], a multithreaded operating system for WSNs. Figure 4 shows the software architecture of the sensor node. A medium access control (MAC) protocol for hop-to-hop communication, a routing component for efficient data transmission, and device drivers for the operation of sensors were additionally implemented on the RETOS kernel to support the monitoring system. The application consists of a multi-hop relay for data transmission, a sensor controller for controlling sensors and reading the sensing value, and a data collector for converting sensing values into physical gas concentration information.

To support straightforward installation and removal, the sensor nodes in the system automatically construct a tree-based source-to-sink routing table. The base station periodically broadcasts hello messages to signal its existence, and the neighbor node that captured the broadcast message registers itself as a child of the base station. Based on the tree topology, each node in the system generates sensing values and forwards them to the host. A simple CSMA/CA MAC protocol is used for the communications. Considering the significantly high energy consumption of the add-on sensor board, the energy efficiency of the base node that contains a radio transceiver is less dependent on the energy characteristics of the underlying MAC protocol.

**Figure 5.**
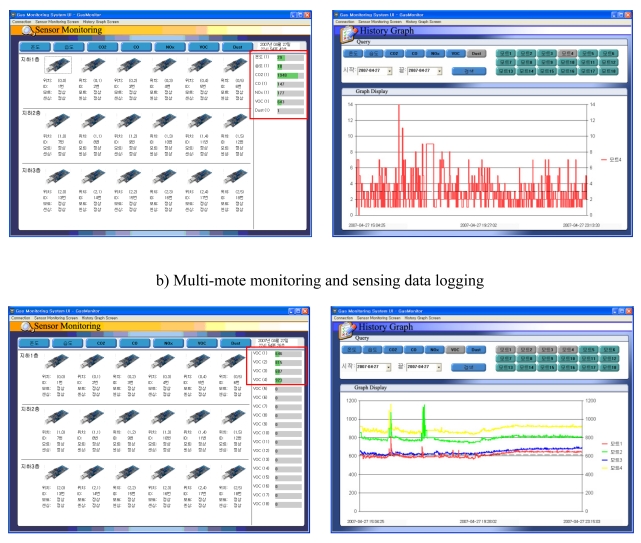
Screenshots of the host PC application. a) Single mote monitoring and sensing data logging b) Multi-mote monitoring and sensing data logging

The application on the host (Figure 5) has two purposes. One is to provide sensing information to users so they can evaluate the performance of the sensors and nodes. The other is to provide a pollution detection alarm to users. We divided the application into two functions: sensor monitoring and a history graph. In the sensor's monitoring window, the application provides real-time sensing information. The information is displayed by either the sensor or node. In the history graph window, the sensing information is retrieved and displayed by a combination of sensors, nodes, and sensing period upon the user's query. Note that the information from all sensors is automatically logged in the base station, and the logging cycle is adjustable.

## 7. Experiments

### 7.1. Experimental Setup

The experimental results discussed earlier in the paper were obtained from the hardware measurements. We inserted a 1-Ω shunt resistor between the battery and the sensor node, and another 1-Ω resistor was soldered to the sensor board to measure the power consumption of each component. Using an Agilent DSO6034A oscilloscope and a Fluke 125 ScopeMeter, we measured the characteristics of the gas sensors. An Agilent 34970A data logger was used to measure the current drawn through the shunt resistor connected to the batteries.

### 7.2. Sensor Validation

**Figure 6.**
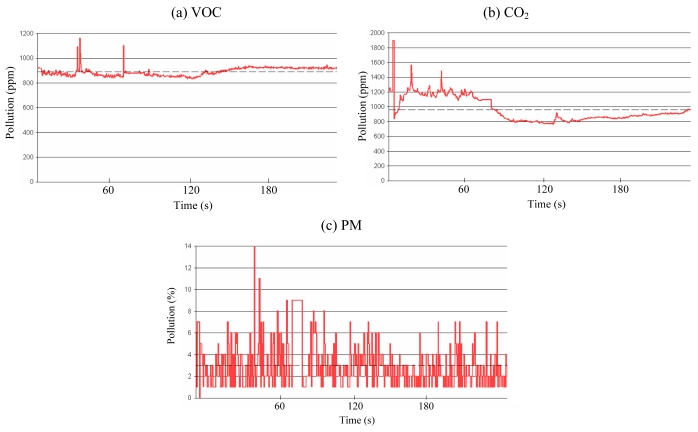
Sensing value changes over time.

The initial experiments were conducted to obtain and understand the basic characteristics of the sensors in the prototype hardware. First, we measured the variation of the sensing data to examine the sensors' stability. One sensor node was placed in a fixed location, and the atmosphere was controlled so that no substance could cause pollution. The participating sensors were the semiconductor-type VOC sensor, the NDIR-type CO_2_ sensor, and the LED-type PM sensor. Figure 6 shows the results of the initial experiments. As shown in Figure 6(a) and Figure 6(b), the VOC and the CO_2_ sensors display stabilized values after a certain period of time because the sensors use heaters to detect pollutants, and the sensing values can be reliably measured only when the component is heated to a certain temperature. On the other hand, the PM sensor is insensitive to time, as shown in Figure 6(c).

**Figure 7.**
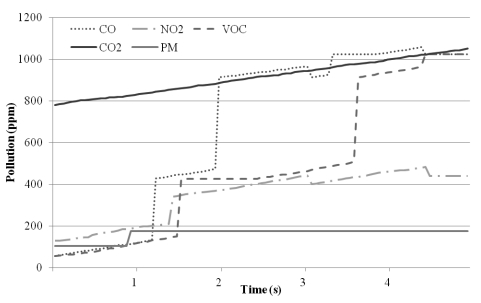
Response to a pollutant (tobacco smoke).

Another experiment was conducted to determine how each sensor responds to polluted air. The experiments were conducted with tobacco smoke that was generated near the sensor board. Tobacco smoke contains multiple pollutants, such as CO, CO_2_, NO_2_, VOCs and so on [21], and also contains particles that the PM sensor can detect. The results are shown in Figure 7. The CO, NO_2_, and VOC sensors responded immediately because tobacco smoke contains high levels of such chemicals. The heating semiconductor sensor also showed a quick response time. At the time of pollution generation, the CO_2_ sensor did not detect the pollutant, but as time passed, the detected concentration of CO_2_ rose. This delay in detection occurred because tobacco smoke contains less CO_2_, and the NDIR-type sensor does not respond as quickly as the semiconductor sensors. In the case of the PM sensor, there was no remarkable detection, since tobacco smoke contains little PM.

### 7.3. Energy Management

All sensor nodes and sensor boards in APOLLO can operate powered by batteries to support flexibility. Therefore, an important requirement is to extend the overall lifetime of the system. The lifetime can be lengthened simply by running the whole system at an extensively low duty cycle. The implementation of APOLLO allows such a low duty cycle to be achieved by exploiting the pulsed mode of the semiconductor chemical gas sensors.

**Figure 8.**
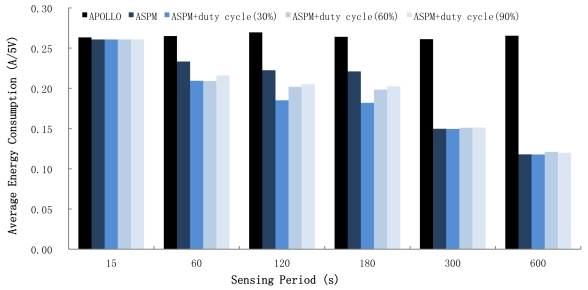
Average energy consumption.

**Figure 9.**
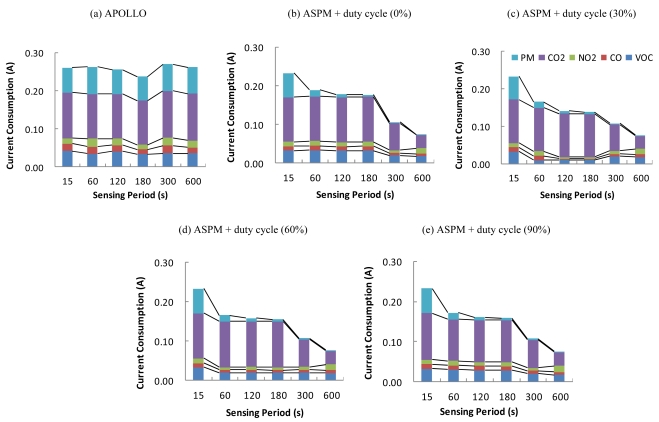
Energy consumptions of each gas sensor.

To evaluate the energy consumption and lifetime of the sensor node, sensing data were generated at each sensor node every 15, 60, 120, 180, 300, and 600 seconds. Figure 8 shows the energy consumption of the sensor board for each case. In the figure, “APOLLO” represents the energy consumption when no power management technique was applied. Each experiment was conducted five times under the same conditions, but only one type of sensor was activated to measure the amount of energy consumed by each individual sensor. The results are shown in Figure 9. Without a power management technique, the energy consumption of the sensor board was considerably high, as shown in Figure 9(a). If the continuous energy consumption was maintained, the sensors stopped operating in the order of CO_2_, PM, VOC, CO, and NO_2_. Figure 9(b) shows the current drawn when ASPM was applied to APOLLO. The power consumption rate was reduced in three steps: 15 to 60 seconds, 180 to 300 seconds, and 300 to 600 seconds. The first step occurred due to the duty-cycled operation of the PM sensor. Note that the power consumption rates of the other sensors do not change. The remaining steps occurred because the sensors' periods of reading became long enough for duty-cycled operation to begin for all sensors.

Figure 9c-e shows the energy consumption rates of each sensor when the pulsed mode is used. In the experiment, the semiconductor gas sensors were activated at the duty cycle of 30%, 60%, and 90%, respectively. For all three cases, power dissipation was reduced by periodically supplying energy. The sensor board's operation was most energy efficient when the duty cycle was 30%. In this case, the lifetime of APOLLO increased by 27%.

The results shown in Figure 8 and Figure 9 indicate how the sensor type would affect energy consumption. The power consumption of the NO_2_, CO_2_, and VOC sensors, which are heating semiconductors, is significantly affected by the duty cycle, and the preheating time is also important. However, the power consumption of the PM sensor depends on the power management policy rather than on the duty cycle or the sensing period. The PM sensor exhibits a low overhead of power transition. Moreover, the sensor is the LED type and generates the sensing value immediately without any required preheating phase. Finally, the CO_2_ sensor is more influenced by the sensing period than the duty cycle because the power consumption for each sensing is relatively high.

**Figure 10.**
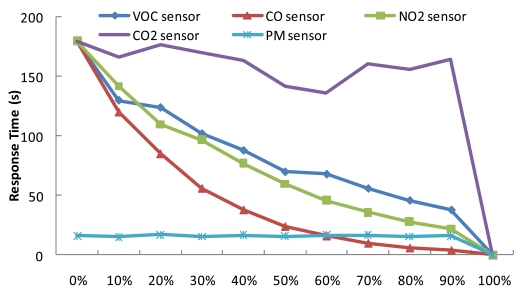
Correlation between sensors' duty cycles and their response times. In the case of semiconductor sensors, average response time is lowered as the duty cycle becomes higher.

Further experiments were conducted to investigate the relationship between the duty cycle and response time for each gas sensor. We measured the response time for each duty cycle with a fixed sensing period of 60 seconds. As can be seen in Figure 10, the response time increases as the duty cycle increases, due to the wake-up time overhead of the gas sensors, shown in Table 3. Depending on its type, each sensor exhibits specific response time characteristics. For example, the LED-type PM sensor does not require a warming-up phase and therefore has a fast response time, whereas the NDIR-type CO_2_ sensor has a long response time.

## 8. Conclusions

Wireless sensor networks that make use of MEMS or micro sensors have enabled a number of sensor nodes to perform intelligent environment monitoring. Previous studies on WSN monitoring have focused on system architecture and networking performance, rather than seeking to understand the characteristics of the various sensors. Generally, MEMS or micro sensors employed in WSNs show a low performance with regards to accuracy compared to the expensive sensors used for precise measurements. In this paper, we developed a prototype air quality monitoring system, named APOLLO, equipped with inexpensive micro sensors to monitor EPA-classified air pollutants. From the design, implementation, and operation of the monitoring system, we were able to understand the characteristics and limitations of the MEMS and micro gas sensors.

The U.S. Environmental Protection Agency's Air Quality Index indicates the levels of major pollutants in the air. An ideal air quality monitoring system measures pollutants according to AQI specifications and reports AQI levels. However, our air quality monitoring system has not been proven to provide legitimate AQI values. As shown in Figure 2, each individual sensor has its own accuracy baseline due to their imprecise manufacturing processes. Moreover, the sensors' sensing values differ from one another, despite being deployed in identical locations. This inaccuracy brings up issues related to the applicability of micro sensors in stringent air quality monitoring applications.

Recent developments in heating, ventilation, and air conditioning (HVAC) systems tend to cover a wide area and include diverse sensing functionality for advanced air quality monitoring and management. Since the HVAC systems focus on detecting changes in air quality [20] rather than measuring the accurate AQI, we believe that an air pollutant monitoring system such as APOLLO is suitable for an automatic ventilation system or HVAC that requires the continuous monitoring of air quality at a low cost.

In future work, we plan to conduct further analysis related to applying MEMS gas sensors to the HVAC systems and develop a technique that improves the efficiency and accuracy of air quality monitoring. In addition, we plan to improve APOLLO to locate the exact area of the contamination sources.

## Figures and Tables

**Table 1.** Air pollutants and their effects on the human body [1,6].

**Category**	**Sources**	**Effects**
CO	gas heaters, leaking chimneys and furnaces, woodstoves, fireplaces, gas stoves	impaired vision and coordination, headaches, dizziness, confusion, nausea
NO_2_	kerosene heaters, unvented gas stoves, heaters, tobacco smoke	eye, nose, and throat irritation, impaired lung function, increased respiratory infections
PM	fireplaces, tobacco smoke, woodstoves, kerosene heaters	eye, nose, and throat irritation, bronchitis, lung cancer
CO_2_	gas heaters, tobacco smoke, woodstoves, fireplaces, gas stoves, automotive products	stimulation of the respiratory centre, dizziness, confusion, headaches, shortness of breath
VOC	paints, paint strippers, aerosol sprays, air fresheners, stored fuels, automotive products, dry-cleaned clothing	eye, nose, and throat irritation, headaches, loss of coordination, nausea, damage to the liver, kidneys, and central nervous system

**Table 2.** Sensor specifications of the prototype.

**Sensor**	**Product**	**Company**	**Size (mm)**	**Range**	**Accuracy**	**Power**	**Type**
CO	MiCS-5521	Micro-Chemical Systems	9.5 × 9.5 × 3.9	10 – 1,000 ppm	-	35 mA/3 V	Heating semiconductor
NO_2_	MiCS-2710	Micro-Chemical Systems	9.5 × 9.5 × 3.9	0.05 – 5 ppm	-	20 mA/3 V	Heating semiconductor
VOC	MiCS-5135	Micro-Chemical Systems	9.5 × 9.5 × 3.9	10 – 1,000 ppm	-	24 mA/3 V	Heating semiconductor
PM	PPD4NS	SHINYEI	69 × 46 × 22	8,000/28 Mℓ	1 μm	90 mA/5 V	LED
CO_2_	D-120	-	55 × 51 × 25	0 – 2,000 ppm	±5.0%	50 mA/12 V	NDIR
Temperature	SHT11	Sensirion	4.88 × 7.24 × 2.5	-40 – 123.8 °C	±0.4 @25 °C	4 mA/3 V	CMOSens

**Table 3.** Wake-up latency and break-even cycle of several sensors.

**Category**	**Wake-up Latency (s)**	**Break-even Cycle (Hz)**
Temp/Hum	0.015	N/A
CO	About 180	About 0.005
NO_2_	About 180	About 0.005
VOC	About 180	About 0.005
CO_2_	About 480	About 0.027
PM	About 360	About 0.015
